# A Review of Synthetic Access to Therapeutic Compounds Extracted from *Psilocybe*

**DOI:** 10.3390/ph16010040

**Published:** 2022-12-28

**Authors:** Raphaël Serreau, Ammar Amirouche, Amine Benyamina, Sabine Berteina-Raboin

**Affiliations:** 1 Unité de Recherche PSYCOMADD, APHP Université Paris Saclay, Hôpital Paul-Brousse, 12 Avenue Paul Vaillant Couturier, 94804 Villejuif, France; 2 Addictologie EPSM Georges DAUMEZON, GHT Loiret, 1 Route de Chanteau, 45400 Fleury les Aubrais, France; 3 Unité de Recherche PSYCOMADD-Psychiatrie Comorbidités Addictions, APHP Université Paris Saclay, Hôpital Paul-Brousse, 12 Avenue Paul Vaillant Couturier, 94804 Villejuif, France; 4 Institut de Chimie Organique et Analytique (ICOA), Université d’Orléans, UMR-CNRS 7311, BP 6759, Rue de Chartres, CEDEX 2, 45067 Orléans, France

**Keywords:** psychedelics, *Psilocybe* mushrooms, tryptamines, psilocybin, psilocin, synthetic access, clinical research

## Abstract

Psychedelics are used for various pathologies of the central nervous system and are currently the subject of much research, some of which relates to the compounds contained in various *Psilocybe*-type hallucinogenic mushrooms. It is difficult, however, to obtain and purify sufficient quantities of these compounds from fungi to carry out biological studies, hence the need to develop simple and efficient synthetic routes. We review here the various syntheses used to obtain these molecules, focusing first on the classic historical syntheses, then the use of more recent metallo-catalyzed couplings and finally the known biocatalytic methods for obtaining these molecules. Other access routes are certainly possible and should be the subject of future research given the therapeutic interest of these compounds.

## 1. Introduction

One of the areas of research on psychedelics that can be used for various pathologies concerns the compounds contained in various *Psilocybe*-type hallucinogenic mushrooms comprising more than 150 species of which about 80 are referenced as hallucinogens [1]. Some other mushroom species also contain these products [2,3,4]. The first two indole derivatives identified in 1958 and then synthesized in 1959 by Hofmann and co-workers were psilocybin and psilocin [5,6]. This current review was conducted following a search, using SciFinder as a database, on methods of synthesizing psilocin or psilocybin. The psilocybin prodrug is rapidly dephosphorylated in the stomach to generate psilocin, which is the active ingredient; however, the latter exhibits instability, particularly in solution [7,8,9,10]. It was shown by various researchers that the main product isolated from mushrooms of the *Psilocybe* genus was psilocybin, although psilocin was also present in significant quantities. Recently, however, two studies showed that, for example, for *P. cubensis* the quantity of the prodrug and the drug were respectively 0.63% and 0.11% of the dry residue in the first study [11] and 1.07% and 0.18% in the second [12]. Overall, the extraction of fresh mushrooms seemed to overestimate the psilocin concentrations in the mushrooms; however, as shown by the results of Hoffmeister [13], the drying process influences the dephosphorylation.

**Figure 1:**
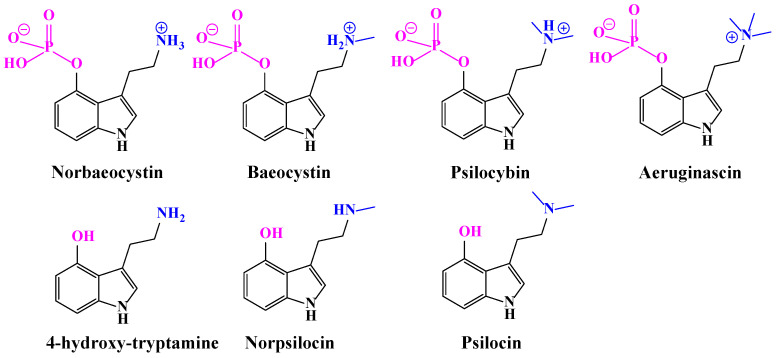
Tryptamines present in genus *Psilocybe* mushrooms.

More recently, Sherwood et al. [14] mentioned that research had mainly focused on psilocin and its prodrug psilocybin [13,15,16,17,18,19,20,21,22], whereas other interesting tryptamines can also be found in lower amounts but have unfortunately not generated much research. Fricke and coworkers [23] described the enzymatic pathways of psilocybin and psilocin. They confirmed that the tryptamines possessing a hydroxy at the 4-position or their phosphorylated analogs with various degrees of *N*-methylation of the aminoethyl chain, known as: 4-hydroxytryptamines, norbaeocystin, baeocystin, aeruginascin and norpsilocin (Figure 1), are also enzymatic substrates.

Only a few rare studies have been carried out on the various analogs present in low quantities in certain species of mushrooms such as *Psilocybe azurescens* and *Psilocybe cyanescens*, in particular, baeocystin and its dephosphorylated analog norpsilocin. Some variable effects following recreational use have been reported during the consumption of some species of mushrooms likely dependent on the proportion of the constituents in the species [11,24].

All of these psychedelics belong to the class of psychoactive substances that act through the activation of serotonin 5-HT_2A_ human receptors as a partial agonist [25,26]. Some of them can also act as 5HT_3_ agonists [27,28]. They generate psychological and physiological effects that are of interest in different pathologies. Phase 2 and 3 clinical trials [29] show favorable results in several CNS indications and the US Food and Drug Administration (FDA) has granted psilocybin Breakthrough Therapy Designation for treatment-resistant depression and the treatment of major depressive disorders [30]. These products, which are particularly interesting in the treatment of depression, need to be obtained under the best conditions in order to be able to carry out the research essential for future clinical use [31]. The number of articles indexed on the subject has grown exponentially over the past 10 years—there have been n = 1520 articles since 1958, more than 50% of which have been published over the past 10 years, showing the dynamics of research on this field.

Other indications include various disorders [32] such as cancer-related existential distress [33], substance addiction [32,34] and obsessive-compulsive disorder [35]. They have also been suggested more recently as a treatment for behavioral disorders and alcohol [36] or tobacco addiction [32,37,38].

Given the increasing prevalence of psychiatric illnesses [39] that can be treated by psychedelics, it seemed of interest to establish an inventory of the synthetic access routes of psilocin and its various derivatives.

## 2. Results and Discussion

### 2.1. Original Hofmann Synthesis and Its Several Improvements

The potential synthetic access routes are the same as those for tryptamine syntheses and yet only a few methods have been implemented to obtain these derivatives.

We will see how these psychedelics are obtained from the indole by substitution of position 3 with a view to inserting the aminoethyl chain or via the creation of the 5-membered ring from a suitably functionalized aromatic. The sequences used depend on the starting synthon and the objective of the synthesis, such as, for example, obtaining labeled molecules or not. A third synthetic methodology involves couplings catalyzed by transition metals, either iridium or palladium.

Among the natural tryptamines treated, the target molecules are listed in Figure 1 and differ only by the degree of methylation of the aminoethyl chain of psilocybin and psilocin.

**Figure 2:**
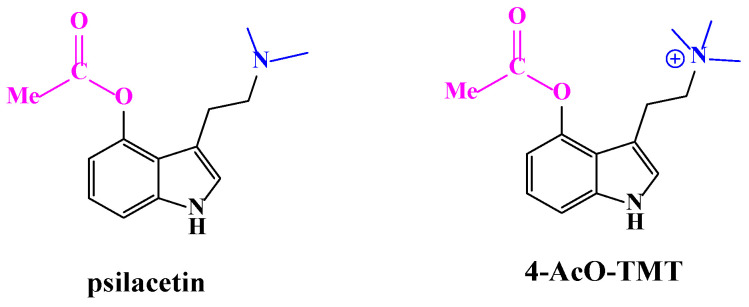
Aeruginascin analog (4-AcO-TMT).

We will also be interested in this review in two analogs: psilacetin (4-acetoxy-*N*,*N*-dimethyltryptamines), which is the acetylated analog of psilocybin and can also be hydrolyzed to active psilocin; and the 4-acetoxy-*N*,*N*,*N*-trimethyltryptammonium (4-OAc-TMT) analog of aeruginascin (Figure 2) [40].

The initial methodology, which is also the most well-documented for obtaining psilocybin, consists in inserting the aminoethyl chain in position 3 of an indole core structure.

**Scheme 1:**
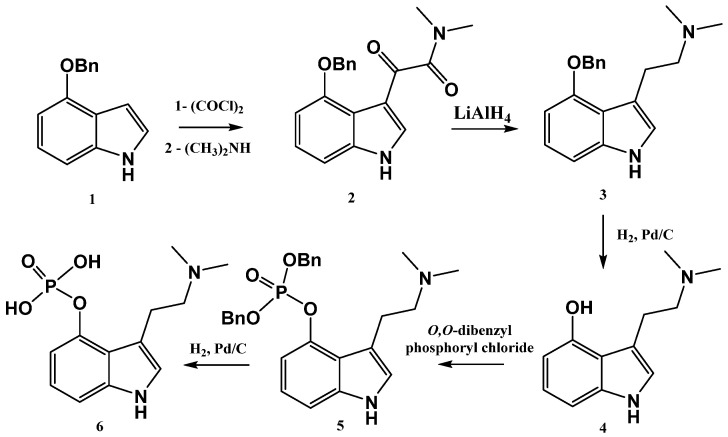
Synthesis of psilocybin by Hofmann et al. in 1958 and 1959 [5,6,41].

Hofmann et al. [5,6,41] developed one of the first syntheses of psilocybin for Sandoz, following the process described in Scheme 1. The 4-hydroxyindole protected by a benzyl group **1** was used as starting material and treated with oxalyl chloride followed by dimethylamine to give the expected stable indole **2**, which was reduced with lithium aluminum hydride to furnish the desired dimethyl aminoethyl side chain **3**. Deprotection of the hydroxyl function using hydrogen with palladium on carbon gave the active psilocin **4**. The prodrug **6** was synthesized by phosphorylation with *O*,*O*-dibenzylphosphorylchloride leading to **5** which underwent hydrogenation on palladium on carbon.

This first synthesis has since been modified by different chemists who have brought many improvements in the yield and purity of the final compound [42,43].

**Scheme 2:**
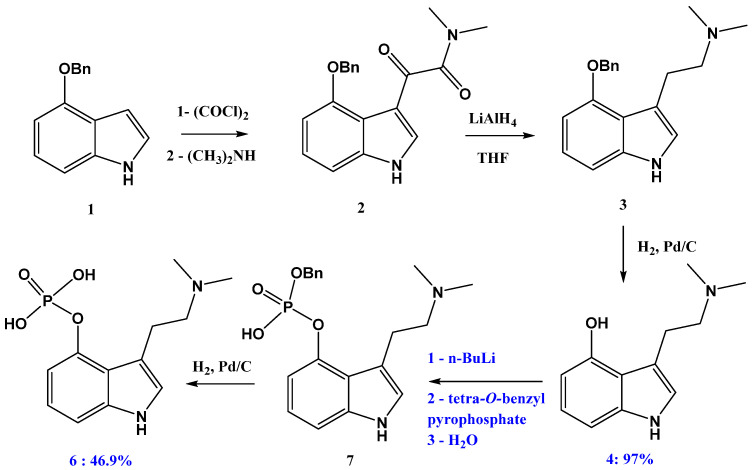
Improved synthesis of psilocybin by Nichols et al. in 1999 [42].

Nichols et al. [2] developed an improved procedure using a different phosphorylation protocol. They accomplished this step with, what they declared was, the most suitable reagent: tetra-benzyl-pyrophosphate which is a stable crystalline reagent compared to the *O*,*O*-dibenzyl phosphoryl chloride used by Hofmann and that led to only 20% yield. With these new conditions, they obtained Psilocybin in 46.9% yield. However, this phosphorylation step was difficult because of the lability of the *O*,*O*-dibenzyl ester. Nichols et al. showed that a hydrolytic cleavage of one *O*-benzyl group occurred at room temperature in the presence of water and the purification of this compound **7** was difficult (Scheme 2).

**Scheme 3:**
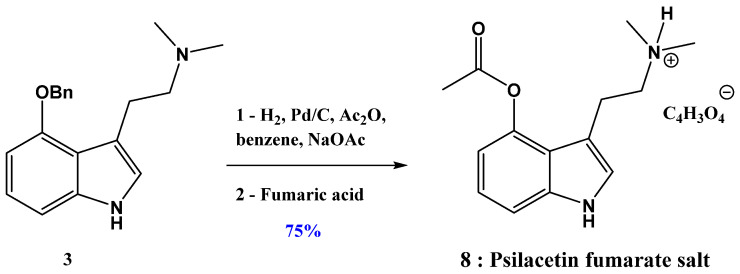
Synthesis of psilacetin (*O*-acetyl)-prodrug by Nichols et al. in 1999 [42].

To overcome these phosphorylation problems, Nichols et al. [42] decided to synthesize the 4-acetoxy-*N*,*N*-dimethyltryptamine by hydrogenation of compound **3** with Pd/C in presence of acetic anhydride and sodium acetate in benzene; then, after filtration of the catalyst and some insoluble salts, fumaric acid was added. This prodrug is much easier to synthesize and uses atom-economical reagents. Psilacetin salt has the advantage of being easily crystallizable (Scheme 3). Psilacetin is, moreover, more stable than psilocybin.

**Scheme 4:**
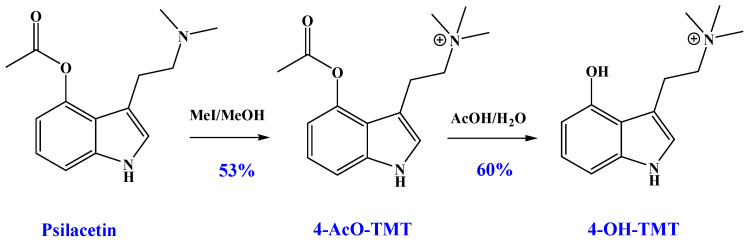
Synthesis of aeruginascin by Manke et al. [40].

Recently, in 2020, Manke et al. [40] reported the first synthesis of a metabolite of aeruginascin, an active compound found in some psilocybes such as *Inocybe aeruginascens* [13,14,21]. This mushroom was described as a source of hallucinations, causing only a euphoric effect without the dreaded bad trip or dysphoric experience [20] that other mushrooms can generate. Unfortunately, the activity of aeruginascin has not been extensively explored [21]. Aeruginascin can also undergo hydrolysis to lead to its active metabolite 4-HO-TMT (4-hydroxy-*N*,*N*,*N*-trimethyltryptamine). The synthesis of this derivative was achieved from psilacetin in the form of the fumarate salt, which was methylated with an excess of iodomethane in methanol to provide 4-acetoxy-*N*,*N*,*N*-trimethyltryptammonium in 53% yield and then hydrolyzed in the presence of acetic acid in water (Scheme 4).

These compounds (4-AcO-TMT and 4-OH-TMT) were recrystallized from water, for the first time, and characterized by single-crystal diffraction. This easy-to-synthesize active metabolite of aeruginascin showed binding at three receptors: 5-HT_1A_, 5-HT_2A_ and 5-HT_2B_ and appeared to be as interesting as psilocybin, according to the authors.

**Scheme 5:**
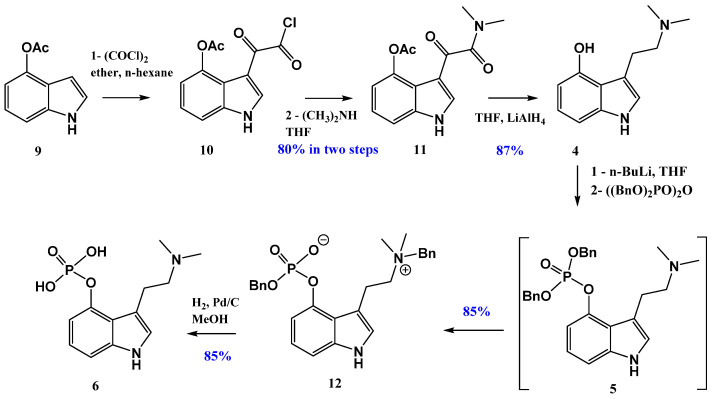
Synthesis of psilocybin by Shirota et al. [43].

Shirota et al. [43] developed the synthesis of psilocin and psilocybin from the 4-hydroxy-indole at the gram scale without the need for chromatographic purification (Scheme 5). They obtained 85% yield and notably showed the spontaneous intramolecular migration of one of the benzyl groups of compound **5** to form the zwitterionic *N*,*O*-dibenzyl phosphate derivative **12.** This rearrangement product was completely characterized by 2D NMR analyses, HMBC and NOESY correlations [43].

More recently, Sherwood et al. [44] described an improved, practical and scalable synthesis according to the Speeter–Antony tryptamine synthetic pathway [45] previously used. As these conditions did not allow them to obtain the same yields, they adapted Shirota’s conditions to obtain psilocybin with high purity (99.9%) by simple trituration and filtration in various solvents as purification processes. The low solubility of the rearrangement product allowed its isolation by filtration.

Using commercially available 4-acetoxyindole **9**, acylation in the presence of oxalyl chloride in ethyl ether requires careful treatment to remove traces of residual oxalyl chloride, which generates the decomposition of the chloro-compound intermediate. Washing this intermediate with heptane makes it possible to obtain a relatively stable compound **10**, as a yellow solid, which can be kept for a few days. However, the reaction with dimethylamine can also be launched directly at the end of the treatment in the presence of dimethylamine in solution in THF. This protocol gave the desired product in 80% yield with a purity of 96%, which can be increased to >99% by recrystallization in isopropyl alcohol.

**Scheme 6:**
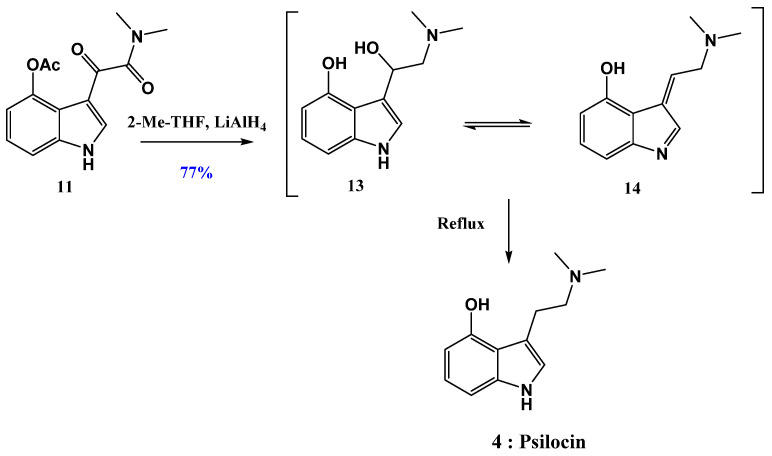
Reduction of ketoamide **11** via β-hydroxy intermediate **13**.

The reduction of compound **11** with LiAlH_4_ in THF at reflux temperature led to a mixture of compounds (Scheme 6) with the β-hydroxy derivative **13**, which can be limited by a prolonged refluxing time, but in this case, decomposition occurred. 2-MeTHF was used because of its higher boiling point and allowed the authors to obtain psilocin **4** in 77% yield and 99.2% HPLC purity after an adapted treatment to remove an excess of LiAlH_4_. Psilocin was described as unstable and partially broken down into colored derivatives ranging from green to purple over time due to oxidation issues, especially if the psilocin was not obtained pure or not stored in a solid state.

**Scheme 7:**
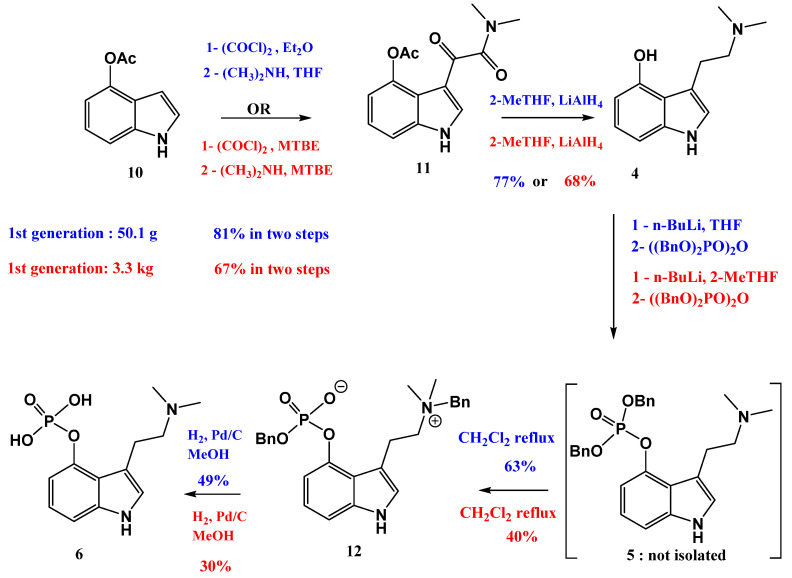
Results of the scale-up for Sherwood’s psilocybin synthesis [44,46].

The phosphorylation of psilocin via the rearrangement protocol leading to the zwitterionic intermediate **12** (Scheme 5) was then also implemented. By varying the reaction temperature and the washing solvents, Sherwood et al. [44] managed to obtain **12** pure in 63% yield. The catalytic hydrogenation of this zwitterionic compound with 10% Pd/C at atmospheric pressure in methanol gave rapid access to psilocybin in the form of a purple solid. A study of the most appropriate solvents for the purification of psilocybin by recrystallization was carried out and the product of interest was obtained pure after three successive recrystallizations with a purity of 99.9% determined by HPLC. The first recrystallization was carried out from acetone containing 30% water, the second from water containing 30% acetone and the last from deionized water. Scaling up from 50 g to 3.3 kg of starting material with some solvent modifications generated a slight decrease in yields at each step (Scheme 7). This level of purity has allowed its use in clinical trials.

**Scheme 8:**
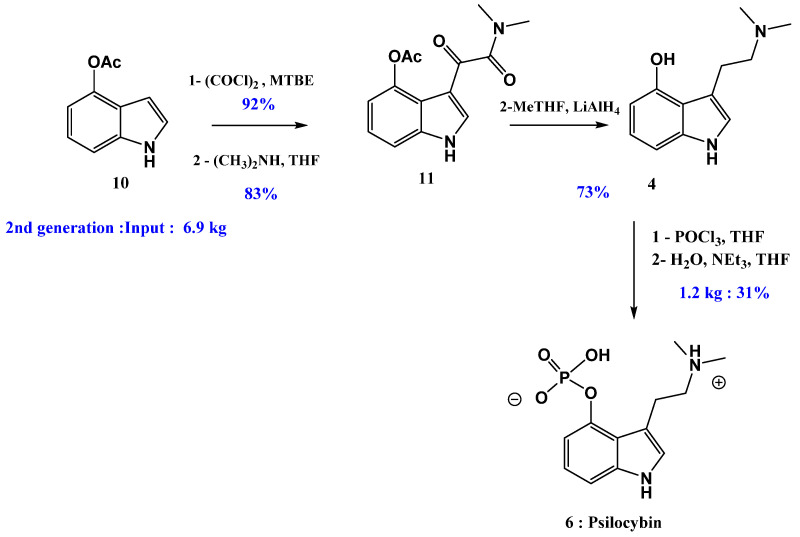
Results using POCl_3_ in the second generation of Kargbo’s psilocybin synthesis [46].

A second-generation synthesis was also developed on a kilogram-scale production by the same team [46] and provided an alternative solution to the use of very expensive reagents such as TBPP tetra-benzyl pyrophosphate, which was replaced, for the first time, by phosphorus oxychloride. This new method of synthesis is a more atom-efficient process for the production of around one kilogram of psilocybin with an overall yield equivalent to that previously obtained on a smaller scale (Scheme 8).

However, the use of POCl_3_ instead of TBPP generates several di-phosphorylated intermediates after aqueous quenching and hot recrystallizations from water or methanol can cause the hydrolysis of psilocybin to psilocin. This phosphorylation step remains the most problematic step of the synthesis.

### 2.2. Synthesis of Isotopically Labeled Psilocin and Other Psilocin Synthesis

**Scheme 9:**
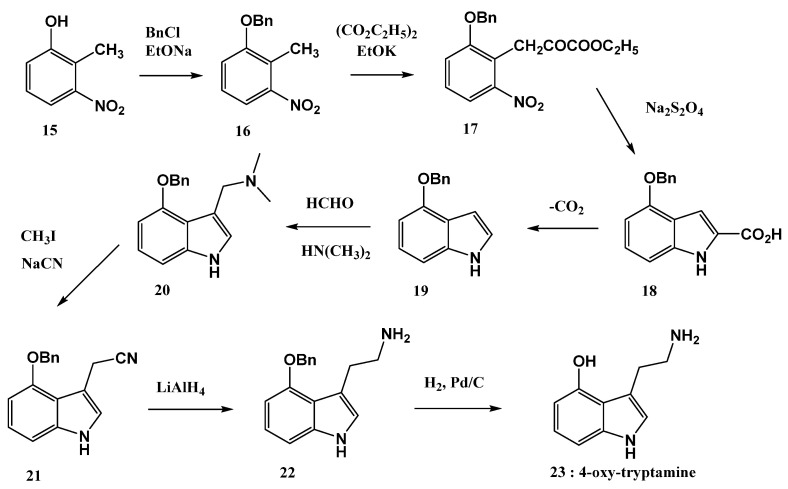
4-oxy-tryptamine synthesis by Hofmann [47].

In 1955, Hofmann et al. [47] described a new synthesis of bufotenin or 5-hydroxy *N*,*N*-dimethyltryptamine and related 4-oxy-tryptamines according to Scheme 9. In addition to bufotenin, compounds (still unknown in 1955) were also synthesized by analogy. This is how two positional isomers of serotonin, 4- and 6-oxytryptamine, were described based on the serotonin synthesis of Hamlin and Fischer [48]. The *N*,*N*-dimethylated 4-oxytryptamine called psilocin was extracted from *Psilocybe* three years later by the same researchers.

**Scheme 10:**
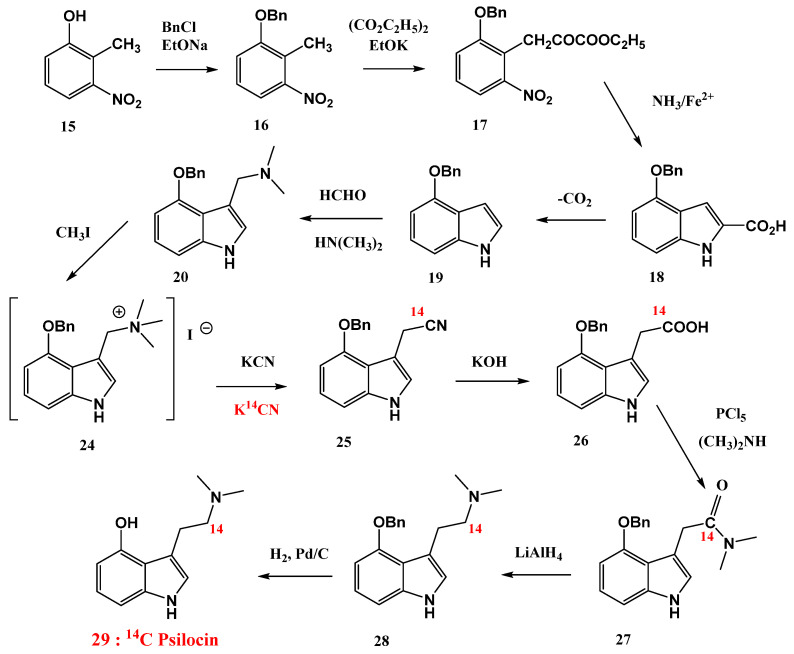
^14^c-labeled psilocin synthesis by Kalberer et al. [9].

**Scheme 11:**
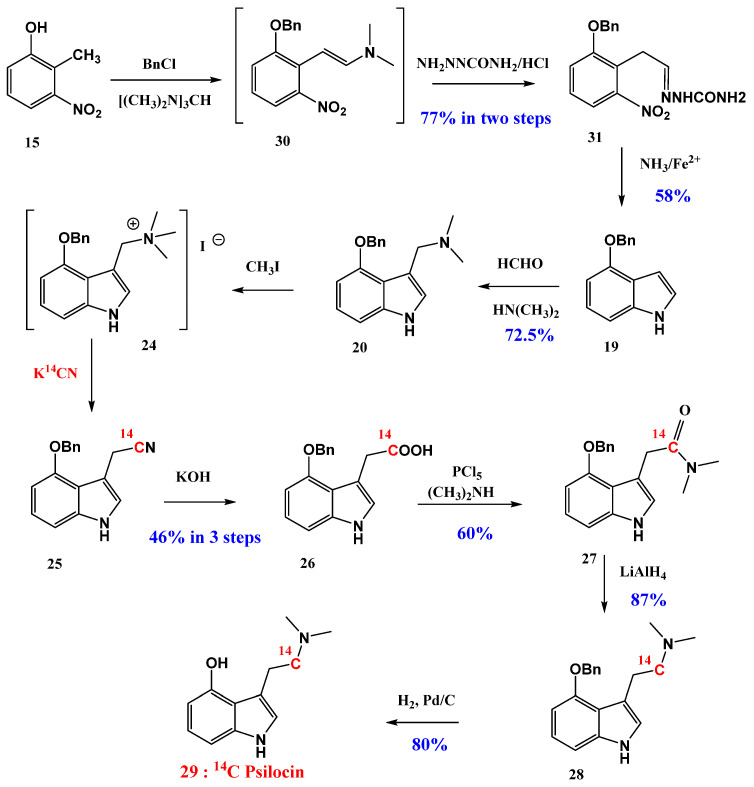
Improved ^14^C-labeled psilocin synthesis by Poon et al. [49].

**Scheme 12:**
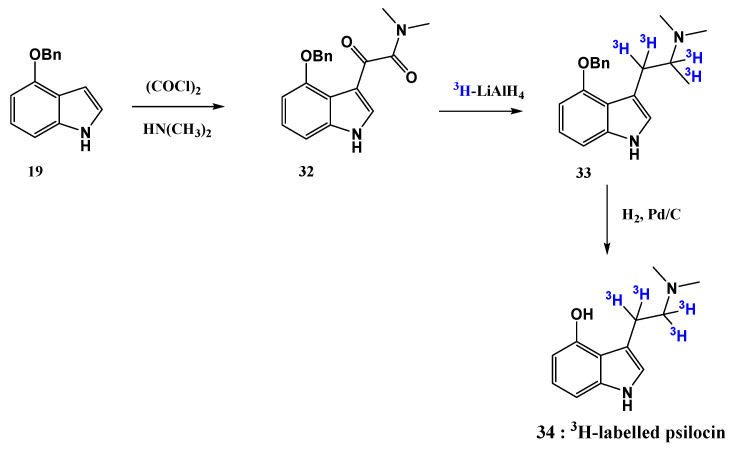
^3^H-labeled psilocin synthesis by Poon et al. [49].

This synthetic method was also used in 1962 by Kalberer et al. [9] using labeled K^14^CN instead of NaCN (Scheme 10) to obtain the ^14^C-labeled psilocin **29**. The previous steps were similar to those previously. This procedure was improved by Poon et al. in 1985 [49] (Scheme 11). Poon also described one procedure applied to the synthesis of ^3^H-labeled psilocin (Scheme 12).

Poon used tris (dimethylamino)methane to generate a non-isolated product that afforded semicarbazone by treatment with semicarbazide hydrochloride by a procedure already described by Kruse [50]. After cyclization in 4-benzyloxyindole **19** then formylation and amination, the 4-benzyloxygramine **24** was heated in presence of K^14^CN at reflux temperature leading to the expected labeled compound after acidification of the solution. Using phosphorus pentachloride, dimethylamine then LiAlH4 as a reductive agent, followed by deprotection of the benzyl group, the ^14^C-psilocin **29** was synthesized in a shorter time and a better yield, 42% yield from **26** compared to Kalberer’s protocol < 10% yield in their hands (Scheme 11). 

The synthesis of ^3^H-labeled psilocin **34** was obtained by treatment with oxalyl chloride followed by the addition of dimethylamine. The use of labeled lithium aluminum hydride (^3^H-LiAlH_4_) followed by debenzylation by hydrogenolysis led to the expected compound in good yields (Scheme 12).

**Scheme 13:**
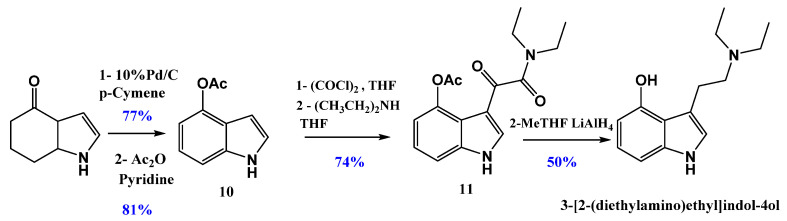
3-[2-(diethylamino)ethyl)]indol-4-ol synthesis by Repke et al. [51].

**Scheme 14:**
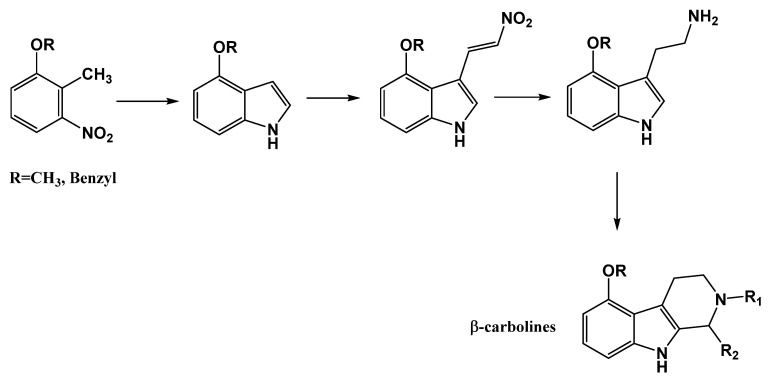
β-Carbolines synthesis by Repke et al. [52,53].

Repke [51,52,53] also investigated the synthesis of these compounds which he pharmacomodulated at the level of the aminoethyl chain (Scheme 13) or by transforming indole into various β-carbolines (Scheme 14). He obtained indole precursors of β-carbolines starting from 2-nitro-6-alkoxy-toluene via the Leimgruber and Batcho method [54]. The tryptamines were synthesized from indole in presence of 1,1-dimethylaminonitroethylene followed by reduction with LiAlH_4_ then a Pictet–Spengler reaction with glyoxylic or pyruvic acids [55] afforded the corresponding β-carbolines.

**Scheme 15:**
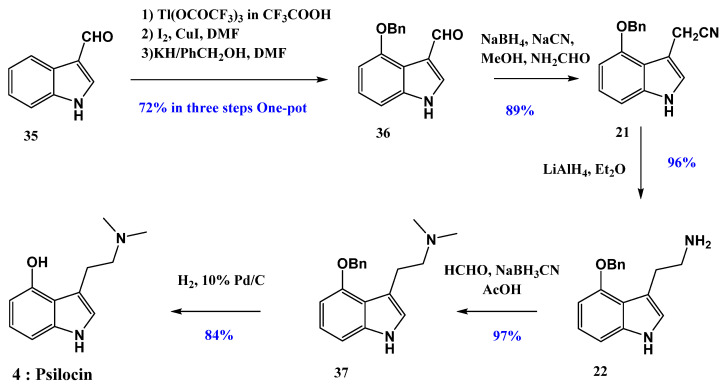
Psilocin synthesis by Somei et al. [56].

In 1998, Somei et al. [56] described a five-step synthesis of psilocin **4** starting from indole-3-carbaldehyde **35** according to the following Scheme 15. In 2002, they applied their strategy to the synthesis of various analogs bearing a formyl or bromine substituent in the 5- or 7-position [57].

### 2.3. Metallo-Catalyzed Psilocin Synthesis

**Scheme 16:**
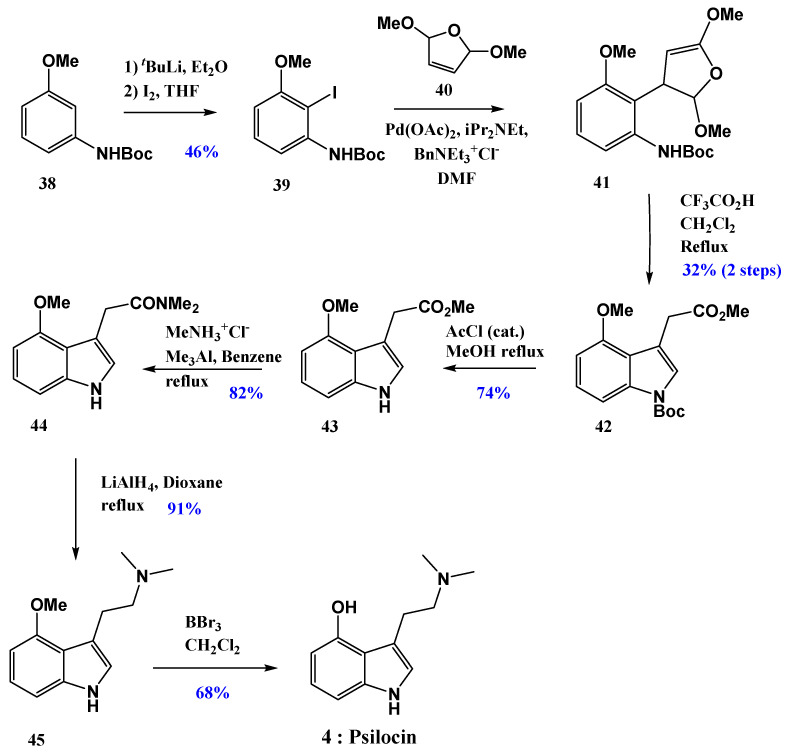
A new synthesis of psilocin by Ogasawara et al. [59].

Metal-catalyzed reactions require catalytic quantities of metal and can be used with a wide range of functional groups, explaining the widespread use of these transformations, especially in indole synthesis. However, only a few examples have been described for tryptamine synthesis. It was in 1999 that Ogasawara reported a new synthesis of psilocin **4** from *N*-tert-butoxycarbonyl-3-methoxyaniline **38** prepared from commercially available 3-methoxyaniline [58]. After iodination of this compound to afford **39** in 46% yield, the team was the first to involve a metallo-catalyzed Heck-type coupling with palladium acetate using 2,5-dihydro-2,5-dimethoxyfuran **40** as the double-bond component. After refluxing in trifluoroacetic acid and deprotection of the Boc *N*-protective group, the authors used the Weinreb conditions [59] to transform the methyl ester **43** into *N*,*N*-dimethylamide **44**. A reduction of the amide with LiAlH_4_ followed by deprotection of the methyl alcohol substituent using boron tribromide afforded psilocin **4** in 5.5% overall yield (Scheme 16).

**Scheme 17:**
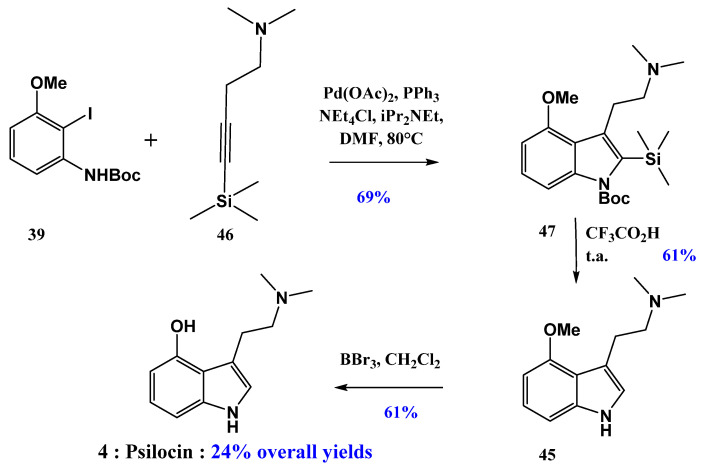
Concise synthesis of psilocin by palladium-catalyzed cyclization [58].

Scammells et al. [60] reported a short preparation of the indole core structure directly via palladium-catalyzed cyclization between a 4-hydroxy *ortho* iodoaniline and a silylated alkyne bearing the dimethylaminoethyl chain **46**. Involving the Larock method [61] where the palladium was inserted on the less hindered site of the alkyne, they were able to generate the desired 3-substituted indole after cyclization **47**. This compound bearing the trimethylsilyl group in the 2-position was deprotected in the presence of trifluoroacetic acid. The third step consisted in removing the methyl group on an alcohol substituent using boron tribromide. Using this protocol, psilocin **4** was synthesized in 24% overall yield (Scheme 17). This synthetic methodology requires the prior synthesis of the substrates used. Thus the alkyne **46** is obtained from 3-butyn-1-ol, in 88% total yield, by a procedure already described [62]. Tosylation followed by *N*,*N*-dimethylation by nucleophilic substitution followed by lithiation of alkyne, and reaction with trimethylsilyl chloride led to the suitably functionalized alkyne **46**. The *N*-*tert*-butoxycarbonyl-2-iodo-3-methoxy-aniline **39** was obtained in three steps from 3-methoxy-aniline protected with a *tert*-butoxycarbonyl ortho-director group followed by a lithiation then consecutive iodination. The expected total regioselectivity of palladium cross-coupling was confirmed by X-ray crystallography.

**Scheme 18:**
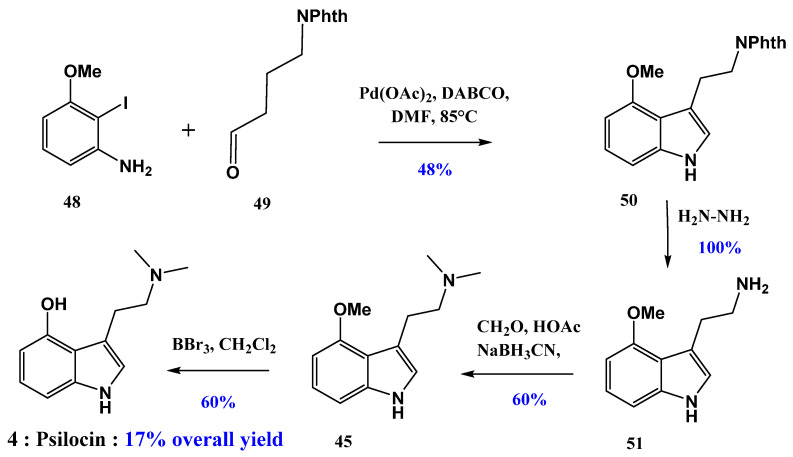
Synthesis of psilocin by palladium coupling [63].

Psilocin was also obtained on a small scale (only 5.6 mg) from 2-iodo-3-methoxy-aniline **48** and phthalimide-protected 4-aminobutanal **49** in presence of palladium acetate and DABCO (1,4-diazabicyclo [2,2,2]octane) in DMF at 85 °C. Jia et al. [63] investigated the scope and limitations of this palladium coupling with various substrates (Scheme 18). Two nitrogen-protecting groups of 4-aminobutanal were evaluated (Boc and Phth) and synthesized from the commercially available corresponding aminoketal. The method was applied to various natural products. For psilocin synthesis, they used the phthalimido-protected 4-aminobutanal **49** with the 2-iodo-3-methoxy-aniline **48** to lead to the indole core structure **50** whose phthalimido group was deprotected in the presence of hydrazine. The resulting amine **51** was dimethylated, then a final deprotection with boron tribromide provided access to the target molecule **4** in 17% overall yield (Scheme 18).

Another alternative is to start from an indole and introduce an alkyl chain in position 3. This is difficult because of the few electrophilic reagents giving access to the aminoethyl chain, whether protected or not. 

**Scheme 19:**
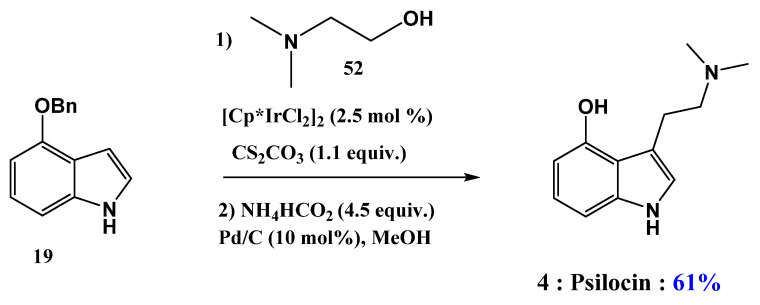
Interesting direct iridium-catalyzed synthesis of psilocin [64].

Bartlucci et al. [64] reported in 2016 an interesting selective iridium-catalyzed alkylation in position C3 of the indole heterocycle with *N*-protected ethanolamine via a borrowing hydrogen procedure. Their first tryptamine examples were successfully obtained after 48 h at 150 °C in presence of [Cp*IrCl_2_]_2_ as catalyst and Cs_2_CO_3_ as base. However, it should be mentioned that some alcohols gave complex mixtures. They described the use of commercially available *N*,*N*-dimethylethanolamine **52** to synthesize psilocin, bufotenin and serotonin in two steps from the 4-benzyl-protected indole **19** or 5-benzyl-protected indole followed by hydrogenolysis of benzyl with NH_4_HCO_2_ and 10% Pd/C in 61%, 67% and 62% yields, respectively (Scheme 19).

These coupling reactions catalyzed by transition metals are very interesting to obtain small quantities of products for analysis but are more difficult to use on a large scale for production due to the availability and high cost of such catalysts. 

### 2.4. Biocatalytic Route

**Scheme 20:**
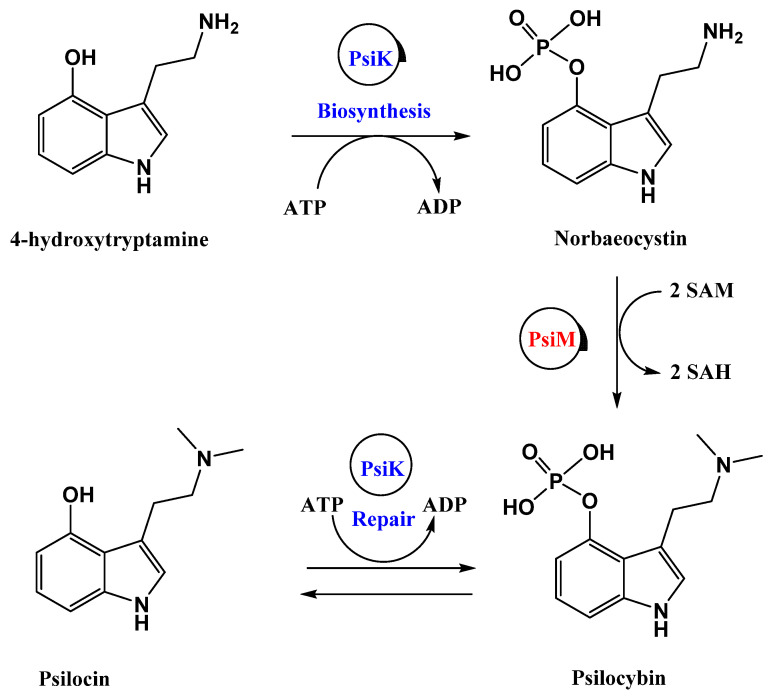
Synthetic/biocatalytic pathway to psilocin [65].

Given, on the one hand, the increasing demand for these active ingredients for use in clinical trials and, on the other, the difficulties encountered in the known synthetic routes described above, Hoffmeister et al. sought to develop enzymatic access to these drugs. As the kinase gene *psiK* had been identified in *Psilocybe* mushrooms, they used it for the biocatalytic synthesis of psilocin in vitro [23,65,66,67] but also in vivo in microbial hosts [68]. In this case, the compounds must be extracted from complex matrices to eliminate cellular dishes with cell lysate or broth. In order to avoid all these problems, Hoefgen et al. and Adams et al. developed a combined synthesis involving the chemical pathway to biosynthesis according to Scheme 20 [68,69]. This new synthetic/biocatalytic route makes it possible to synthesize the target molecule on a large scale. In these conditions, 893 mg of psilocybin was obtained from psilocin in 88.5% yield [65]. This process eliminates steps that may involve heavy metals and the difficult phosphorylation step that requires expensive reagents. They first produced PsiK using E. coli KRXVpJF23 and studied its properties in vitro. Subsequent work confirming the dual biosynthetic and protective role of PsiK, which accepts 4-hydroxytryptamine but also psilocin as substrates, allowed them to use the repair reaction for their study. In *Psilocybe* mushrooms, an *S*-adenosyl-L-methionine (SAM)-dependent *N*-methyltransferase acts twice after the phosphorylation step to complete the biosynthesis of psilocybin from 4-hydroxytryptamine.

These recent results should promote studies on bioprocesses in order to produce this drug but also other drugs at a reasonable cost and in a greener way. Synthesizing in an eco-compatible manner, new, more stable derivatives that retain the same bioavailability would be a major advance in the field.

## 3. Conclusions

Efficient methods are now available to achieve the synthesis of the psilocybin prodrug, which is usually synthesized because psilocin is described as unstable in solution. However, other prodrugs are possible and easier to synthesize, such as psilacetin, for example. Several studies report the use of other protections and are the subject of recently filed but not yet published patents. Other synthetic methods to access tryptamine analogs have not yet been implemented for these drug candidates due to the lack of commercial accessibility to the starting synthons or due to their difficulty of access; in particular, other metal-catalyzed couplings, although the use of metals in the synthesis route should only be used at the start of the synthesis to avoid the associated toxicity problems. Indeed, some starting building blocks cannot be obtained in a regioselectively pure form, thus requiring purifications that are sometimes difficult and very atom-consuming. The biocatalytic route is a very interesting alternative. However, it is important that further studies be conducted on the accessibility of these compounds. Currently, there is a strong interest in psychedelics. Clinical studies that target various pathologies are underway and should lead to the medical use of some of these substances.

## Data Availability

Data sharing not applicable.
